# Employers’ perspective of workplace breastfeeding support in Karachi, Pakistan: a cross-sectional study

**DOI:** 10.1186/s13006-016-0084-7

**Published:** 2016-09-06

**Authors:** Jamil Ahmed Soomro, Zeeshan Noor Shaikh, Tennegedara Buhary Saheer, Suhail Ahmed Bijarani

**Affiliations:** 1 Department of Community Medicine, Isra University, Hala Naka Road, 71000 Hyderabad, Pakistan; 2 Department of Community Medicine, Dow University of Health Sciences, Karachi, 74200 Pakistan; 3 Department of Nursing and Health Promotion, Oslo and Akershus University College, Oslo, Norway

**Keywords:** Breastfeeding, Working mother, Workplace, Breastfeeding support, Employer

## Abstract

**Background:**

Breastfeeding is considered to be an important measure to achieve optimum health outcomes for children, women’s return to work has frequently been found to be a main contributor to the early discontinuation of breastfeeding. The aim of the study is to assess workplace breastfeeding support provided to working mothers in Pakistan.

**Method:**

A workplace based cross-sectional survey was conducted from April through December 2014. Employers from a representative sample of 297 workplaces were interviewed on pre-tested and structured questionnaire. The response rate was 93.7 %. Prevalence of workplace breastfeeding facilities were assessed in the light of World Alliance for Breastfeeding Action (WABA) guidelines.

**Results:**

Among non-physical facilities, all workplaces offered 3 months paid maternity leave, 45 % of the sites were offering task adjustment to mothers during lactation period. Only 15 % of the sites were offering breastfeeding breaks to working mothers. Physical facilities that include a breastfeeding corner, refrigerator for storing breast milk, breast milk pump and nursery for childcare were provided in less than 7 % of the sites. Multinational organizations provided better support compared to national organizations.

**Conclusion:**

Support for continuation of breastfeeding by working women at workplaces is inadequate; hence, women discontinue breastfeeding earlier than planned. Policies need to be developed and enforced, employers and employees need to be educated and supportive environment needs to be created to encourage and facilitate breastfeeding friendly worksite environment.

## Background

Breastfeeding is the best way of providing essential nutrients to infants for their optimum growth and development [1]. In many developing countries, most of the children deaths occur during the first year of life [2]. Pakistan ranks second, after Afghanistan, in child mortality in Asia [2, 3]. Most of these deaths can directly and/or indirectly be attributed to nutritional deficiencies in early stages of life. Increasing population, poverty, illiteracy, unemployment and sociocultural structure are leading root causes of nutritional deficiencies in children. In Pakistan, parents do not earn enough to provide quality food for their children [3].

Breastfeeding is a cost-effective way of saving young lives and proved to have a direct relationship in reducing under-five children’s mortality [3, 4]. According to UNICEF, 22 % of neonatal deaths in Pakistan could be prevented by initiating breastfeeding within the first hour of birth, while 16 % of infant deaths could be avoided if breastfed from day one [4]. The prevalence of breastfeeding up to 1 year in Pakistan from 1983 to 2008 has declined from 96 to 31 % [5]. A woman’s return to work has frequently been found to be a main contributor to the early discontinuation of breastfeeding [6, 7].

Pakistan Demographic and Health Survey (PDHS-2007–2008) indicated that 63 % of the infants less than 6 months were bottle fed and the majority of their mothers were reportedly employed [8]. Lack of workplace support for both physical and non-physical roles resulted in partial or complete discontinuation of breastfeeding by mothers who resumed work after delivery [6, 9].

In urban areas of Pakistan, most of the women cannot afford to stay home longer because they serve as an important contributor to their family income. Within 2 to 3 months after delivery, they are expected to resume their work and perform the same tasks. The lack of a supportive environment for breastfeeding can result in early discontinuation of breastfeeding. A qualitative study in Pakistan reported workplace barriers as one of the main reasons that results in early cessation of breastfeeding among working mothers [10].

Extensive evidence is available about direct relationship of breastfeeding support at the workplace and continuation of breastfeeding in working mothers [6, 10–12].

Breastfeeding facilitation at workplaces is productive for not only employees but also employers. Productivity is social as well as economic. Support results in retention of employees and reduced staff turnover. Breastfeeding support during work hours resulted in higher percentage of working mothers resuming work. Moreover, it also resulted in earlier return than those who were not provided a supporting environment [6, 10–12]. Employees felt more affiliation and ownership of the organization and committed extra effort and hours [1, 13, 14].

World Health Organization, International Labour Organization and World Alliance for Breastfeeding under the theme of “breastfeeding and work, let’s make it work” have adopted a set of guidelines for the employers to facilitate mothers to successfully combine breastfeeding with work. These guidelines include respect for national laws on paid maternity leave, provision of breastfeeding breaks, breastfeeding corner, nursery for childcare, flexible working hours and also breastfeeding options at work such as return part time [1, 15, 16]. The available local studies [5, 10] suggest the need for more observational studies to investigate the status of workplace breastfeeding facilities. The purpose of the present study was to assess the workplace breastfeeding facilities.

### Definition of terms

The employer: the head or a person representing him or her in a workplace.

The working mother: working women after delivery to 2 years of the lactation period.

Physical breastfeeding facilities: include breastfeeding corner, refrigerator, breast milk pump, jobsite crèche.

Non-physical breastfeeding facilities: include breastfeeding breaks, maternity leave, task adjustment, breastfeeding options.

National organization: the workplaces with the local employer from Pakistan.

Multinational organization: the employer from any country other than Pakistan.

According to WABA guidelines [15], breastfeeding breaks refers to the provision of breastfeeding breaks to working mothers for minimum 1 h in a shift of 6–8 h. Maternity leave: three months paid maternity leave according to the national guidelines [16]. Breastfeeding corner: a locked room in the organization allocated for lactating mothers. A place for storing breast milk: a refrigerator ideally placed in a lactation room identified for storing mother milk. Jobsite crèche: a jobsite nursery where babies are cared during working day. Task adjustment or lighter job: **t**he mother is transferred to a place near her home to facilitate with her breastfeeding or mother in a similar site offered a lighter job after her return to work during the lactation period. Provision of information regarding breastfeeding options: Mothers are provided information regarding breastfeeding options upon their return to work such as return part time, extended maternity leave and information regarding availability of breastfeeding facilities i.e. breastfeeding breaks, breastfeeding corner, task adjustment etc.

## Method

We conducted a cross-sectional study on 297 randomly selected workplaces of the city of Karachi, the largest city in Pakistan. Karachi has a population of around 23.5 million consisting of people belonging to different ethnicities, social and economic classes [15]. The study setting represents nearly every social class and ethnic group living in Pakistan, moreover, it is home to Pakistan’s huge economic and educational activity [15].

The sampling frame comprises of 2983 workplaces, that include all the hospitals, banks factories and schools registered with concerned government authorities were considered as the relevant study population for the current study [16–19]. The employer was considered the most knowledgeable person in an organization to know about the breastfeeding facilities at work. Therefore, we considered interviewing employers from each sampling site (workplace). The study population (workplaces) was unequal in size, therefore stratified sampling was used. The sample size was estimated based on the prevalence of 26 % of the workplaces had been offering breastfeeding facilities from a similar study in Hong Kong [20]. We used this prevalence because to our knowledge, this was the first time such a study was conducted in Pakistan. Therefore, the sample size was estimated using the formula;

$$ n=\frac{z_{\alpha /2}^2\times p\left(1-p\right)}{\varepsilon^2} $$

Here, α is the desired level of significance (typically 1.96 for a 95 % confidence interval) and *ε* is the margin of error, which I chose to be for this study.

$$ n=\frac{1.96^2\times 0.26\times \left(1-0.26\right)}{0.05^2}=295.6 $$

Approximately, a total sample size of 296 participants was required. Some 11 employers refused to participate in the study and nine workplaces didn’t meet inclusion criteria. So we included other 20 sites to complete our sample size.

Four female Research Assistants with medical background were recruited and trained. Training conducted on objectives of the study tool and data collection procedures and Research Assistants were also involved in pretesting of the questionnaire.

The participants were provided with the information sheet containing details about survey and contact details of the principal investigator.

The structured questionnaire was designed in English and Urdu based on guidelines of World Alliance for Breastfeeding Action [15]. For maintaining the consistency of the study tool, it was again translated to English by two independent health professionals. First three authors having relevant background and an expert (Professor of Epidemiology) at Section for International Health, University of Oslo agreed on contents of the questionnaire. The pretesting of the questionnaire was done on 16 workplaces equally representing private and public sector. The pretesting was done in workplaces of Hyderabad district, which was not included in our study settings. The necessary changes were made in language and style of the questionnaire to improve comprehensibility.

The questionnaire consists of three sections. The first section enquired about sociodemographic characteristics of workplaces: site level (national versus multinational), job site (bank, school, hospital and factory) and type of employer (Government, private, self-employed). The study was focused on the assessment of workplace breastfeeding facilities, therefore, to avoid expected ethical issues, the identifiable information regarding workplaces such as name of site, address and personnel details of employer were not collected.

The second section enquired about the status of breastfeeding supporting environment that included physical facilities such as breastfeeding corner, place for breast milk storage, jobsite crèche (nursery), the non-physical facilities includes breastfeeding breaks, paid maternity leave, lighter jobs and provision of breastfeeding option to mothers by employers on their return to work. The responses were yes or no.

The third section enquired about opinion of employers with regards to supporting breastfeeding practices at workplaces and reasons for missing breastfeeding facilities. Both were open ended questions and answers were later analysed. The common responses were categorized later.

Data were edited and coded manually before entering in to computer software. Double entry was done to reduce the data entry error. The data were analysed on Stata version 13 and SPSS version 22. All the variables were categorical. Therefore, descriptive statistics were used. Breastfeeding facilities variables were presented with frequencies and percentages using charts. Stata version 13 was used to compare the proportion- differences of breastfeeding facilities by type of demographic factors such as type of employer (government/ private) and site level (national / multinational) with 95 % confidence interval. Statistically significant was considered when *p* < 0.05.

## Results

Table 1 shows the characteristics of the sample. Total 317 workplaces were accessed for surveying the breastfeeding facilities, and 297 employers met the inclusion criteria and consented to provide information. The response rate was 93.7 %. The majority of the sample represents banks (42 %), private employers (75 %) and national sites (76 %).

**Table 1:** General characteristics of the sample

Characteristics	Count	Percent %
No of sites	297	100
Banks	123	42
School	87	29
Factories	66	22
Hospital	21	7
Type of employer	297	100
Private	222	75
Government	75	25
Site level	297	100
National	226	76
Multinational	71	24

Table 2 outlines the provision of breastfeeding facilities at work. Among non-physical facilities; employers from all workplaces (*n* = 297) stated that they provide at least 3 months paid maternity leave to mothers before or after delivery as wished by the mother. During a work shift of 6–8 h, at least 1 h in total of paid breastfeeding breaks were offered in 45 (15 %) worksites. Job adjustment in the form of a lighter or safer job was practiced in 134 (45 %) sites during lactation period. It was observed that most of the job assignments for female employees were fixed, with the exception of banks and schools, no other site had franchises or branches to relocate mothers to a workplace closer to their home to facilitate breastfeeding practices.

**Table 2:** Available workplace breastfeeding facilities

Workplace breastfeeding facilities	Employer (*n* = 297)	
	Yes	
	*n*	%
Physical facilities		
Breastfeeding corner for breastfeeding	17	6
Jobsite crèche for childcare	5	1.7
Breast milk pump	3	1
Place for storing breast milk	0	0
Non-Physical facilities		
Paid maternity leave	297	100
Lighter job or task adjustment	134	45
Provided breastfeeding options	63	21
Breastfeeding breaks	45	15

Physical facilities such as a breast milk pump, refrigerator, breastfeeding corner and jobsite crèche (nursery for child care) were available at less than 7 % of the sites.

We compared the status of breastfeeding facilities in public sector with private sector. Only public workplaces (4 %) were reported to provide breast milk pump to working mothers. None of the workplaces had a designated storage place for breast milk see Table 3. There was no statistically significant difference in the workplace breastfeeding facilities between public and private sector such as breastfeeding breaks, breastfeeding corners, onsite job crèche for childcare, task adjustment and provision of information regarding breastfeeding options such as return part time, change in work shift and maternity leave extension.

**Table 3:** Comparison of the provided breastfeeding facilities between public and private sectors

Workplace breastfeeding facilities	Public (*n* = 75)		Private (*n* = 222)		Test of proportion difference (95 % Cl)	*P*-Value
	Yes		Yes			
	*n*	%	*n*	%		
Physical facilities						
Breastfeeding corner for breastfeeding	5	6.7	12	5.4	1.24 (−5.11, 7.64)	0.684
Jobsite crèche for childcare	3	4	2	0.9	3 (−1.5, 7.7)	0.071
Breast milk pump	3	4	0	0	4 (−0.43, 8.4)	0.003
Place for storing breast milk	0	0	0	0	–	–
Non-physical facilities						
Paid maternity leave	75	100	222	100	0	0
Lighter job or task adjustment	36	48	98	44	4 (−9.2, 17)	0.562
Provided breastfeeding options	11	15	52	23	−8.75 (−18.5, 0.99)	0.109
Breastfeeding breaks	10	13	35	16	2 (−11, 7)	0.6115

Overall, multinational sites were providing more breastfeeding facilities compared to national sites. As indicated in Table 4, there was a statistically significant difference (<0.001) in proportions of the workplace breastfeeding facilities among multinational and national sites in the area of breastfeeding breaks of at least 1 h in total for each shift to facilitate mothers to breastfeed or express and store breast milk for later use. Multinational vs national (38 % vs 8 %); breastfeeding corners (13 % vs 3 %) for mothers to maintain their privacy during breastfeeding or expression of breast milk, provision of lighter job or transferring mother to a safe workplace during lactation (63 % vs 39 %) and provision of information to working mothers by employers upon their return to work regarding different options to facilitate mothers to combine breastfeeding with the work (49 % vs 12 %). It was discovered that most of the multinational sites were offering even more than 3 months paid leave, however, we only considered at least 3 months paid maternity leave as per the national guidelines.

**Table 4:** Comparison of the provided breastfeeding facilities between national and multinational sites

Workplace breastfeeding facilities	Multinational (*n* = 71)		National (*n* = 226)		Test of proportion difference (95 % Cl)	*P*-Value
	Yes		Yes			
	*N*	%	*N*	%		
Physical facilities						
Breastfeeding corner for breastfeeding	9	12.7	8	3.5	9.1 (1.3, 17.2)	0.004
Place for storing breast milk	0	0	0	0	0	0
Breast milk pump	0	0	3	1.3	−1.32 (−2.8, 0.16)	0.329
Jobsite crèche for childcare	0	0	5	2.2	−2.2 (−4.1, −0.3)	0.206
Non-physical facilities						
Paid maternity leave	71	100	226	100	0	0
Lighter job or task adjustment	45	63	89	39	24 (11.1, 37)	<0.01
Provided breastfeeding options	35	49	28	12.4	37 (24.5, 49)	<0.01
Breastfeeding breaks	27	38	18	8	30 (18, 42)	<0.01

The workplaces (factories, banks, hospitals and schools) were compared to each other with regards to breastfeeding facilities, in order to see the differences in proportion of facilities provided by different types of organizations. The lighter job or task adjustment of women worker to a safe place during her lactation period was provided by 137 (45.1 %) workplaces, which comprises of 71 (24 %) banks, 27 (9.1 %) factories, 27 (8.8 %) schools and 10 (3.4 %) hospitals. There was statistically significant difference (*p* = < 0.001) in breastfeeding facilities by type sites. Overall breastfeeding breaks were offered by 45 (15.2 %) sites of which 28 (9.4 %) were schools, 8 (2.7 %) hospitals, 6 (2 %) factories and 3 (1 %) workplaces were banks. The difference between various sites was statistically significant (*p* = < 0.001).

Of the 17 workplaces (5.7 %) which allocated a breastfeeding corner for mothers to practice breastfeeding, 7 (2.4 %) were schools, 4 (1.3 %) were factories, 3 (1 %) sites reported were banks and hospitals. The difference between types of workplace was not statistically significant. However, employers from all sites reported to offer 3 months maternity leave. Whereas, none of the employer allocated place for storing mothers milk.

The employers’ perspective about supporting breastfeeding practices at workplaces through open-ended question revealed; 39.7 % (118) employers perceive breastfeeding practice at work as a healthier activity. The participants representing mainly hospitals and schools especially those in the public sector stated that providing supportive environment to working mother would reduce mothers’ absenteeism from work due to baby related sickness 17.8 % (53) and few employers said that it reduced stress of the mothers and increased her performance 12.1 % (36). Thirty one percent employers (93) mainly from private sector of national sites (workplaces) held the government responsible for sponsoring or running lactation programs at workplaces. Many respondents, 27.6 % (82) mentioned that it is a religious obligation. A number of employers representing mainly the private sector were reported to have a negative opinion on breastfeeding support at work such as; consumption of time allocated for work (26 %), financial burden on organization (22 %) and some believe it’s against organization decorum or not seen in a favor of mothers right to breastfeed at work (13 %).

The workplaces reported to have a missing physical or non-physical breastfeeding facility, were further asked to explain the reasons for not providing a particular support (multiple responses were permissible). The data revealed that 52 % of employers are of the view that they don’t know what to do or don’t find any national guideline or policy. Many employers held mothers responsible for not seeking or asking for required support from them (46 %). The employers representing mainly the private sector, considered breastfeeding at work a women personal activity (42 %) and the organization has nothing to do with it. Numerous employers from the banks and private schools think a shortage of space hinders them to facilitate mothers to successfully breastfeed at work (25 %). Twenty four percent of employers predominantly representing private sector, believe supporting mothers lose their concentration or focus from work they are paid for. Few employers (6 %) were not in favour of providing supporting environment to working mothers due to the multiple reasons’ such as; nature of job (banks with high influx of people, shortage of a space/area, rigid working hours), culture issue (embarrassment with breastfeeding at work), very poor female representation (1–3 females over 1000’s males in an organization).

## Discussion

The purpose of this study was to assess the workplace breastfeeding facilities. To the best of the researchers’ knowledge, this study is the first quantitative study conducted in an urban area of Pakistan, focusing exclusively on assessment of workplace breastfeeding facilities using a large random sample. This study reveals that employers’ do not provide supportive environment at workplace for breastfeeding except for paid 3 months maternity leave. The breastfeeding support was slightly better in the multinational sites compared to the national sites.

The findings of this study will enable policy makers to be informed about the status of the existing breastfeeding support provided by employers in the urban areas of Pakistan. Also to revise policies according to the international guidelines, in order to better address the gradual decline in breastfeeding among urban employed women [8, 10].

It is widely believed that, the provision of paid breastfeeding breaks at work increases chances of breastfeeding by working mothers. An urban based qualitative study in Pakistan showed that the job flexibility including breastfeeding breaks at workplace settings were important to working mothers for continuing breastfeeding while employed [21]. In our study, employers from 15 % of the workplaces claimed to provide working women with breastfeeding breaks to breastfeed their babies or express milk. At the policy level in Pakistan, there are no explicit guidelines for employers about breastfeeding support.

A study by Heymann et al., indicated that the rate of exclusive breastfeeding of children under 6 months of age was 9 % higher in countries that guaranteed paid breastfeeding breaks at workplace [22]. However, our findings are not consistent with these many western studies. There can be several reasons for this: existence of workplace breastfeeding policies, lactation programs and additional support provided to mothers through awareness, education and resources in those countries [23, 24]. Our study also found that all workplaces were providing the women at least three months paid maternity leave as per the national policy of Pakistan [10, 16], which suggests that policy support for breastfeeding is vital for improving breastfeeding practices in Pakistan. Results of our study related to maternity leave were consistent with similar studies undertaken in Malaysia and India (93 and 93 %) [11, 25].

Various studies in Western countries suggest that employers provide information to mothers (on their return from maternity leave) about ways they can continue breastfeeding at workplace. This information constitutes areas such as access to facilities to express and store breastmilk, flexible working hours and information regarding other possible options such as return part time, extended maternity leaves [1, 4–6, 9]. In our study employers’ from 21 % of the sites reported to provide information about breastfeeding options upon their return to work. A lack of a national level policy on breastfeeding and lactation programs in our study setting, limits the comparison of the findings with other related studies in different settings [12, 22–24]. Lighter job or flexible work options and task adjustment were consistently identified as the main factors that promote workplace breastfeeding practices [1, 5, 10–12]. In our study employers from 45 % of the sites claimed that they provide lighter job (including reduce working hours) or task adjustment to relocate mothers to a better place to encourage breastfeeding. Our study findings are similar to a study on determinates of workplace support in Malaysia, where a lighter job was provided in 53 % of workplaces and are different from a study conducted in USA [11, 26]. The difference could be due to the difference in study participants, method and different source of the data (secondary type of data) compared to the data used (primary data) in our study.

Numerous studies have reported that a breastfeeding corner or room for maintaining privacy during breastfeeding or expression of breast milk plays a key role in promoting breastfeeding practices at workplaces. A qualitative study in urban settings of Pakistan indicated that the presence or absence of physical facilities, including breastfeeding corner or room for maintaining privacy, can affect a mothers’ decision to continue or discontinue breastfeeding. Results in our study showed that only 6 % of the sites had a designated breastfeeding corner. However, in contrast to this finding, Weber et al. and Kosmala-Anderson et al., have shown a separate room for breastfeeding, 19 % and 15.9 % respectively, in their respective studies [27, 28]. The difference could be due to differences in study population and settings, i.e. the previous studies considered specific workplaces such as factories and hospitals, in contrast our sample mostly comprised of banks and schools.

Our study also explored the employer perspective/opinion of supporting breastfeeding practices at work. The employers came up with multiple responses to an open ended question. Nearly half of the employers indicated that the breastfeeding is the healthier activity i.e. it provide lifelong immunity, contains antibodies, increases maternal bonding, reduces illnesses, is the best nutrition for an infant, health staff recommended it, reduces child mortality, increases life expectancy and quality of life etc. The numbers of respondents also believe that providing a supportive environment to working mothers would lessen mothers’ absence from work due to infant related sickness (18 %), while 12 % of employers think that breastfeeding would reduce the stress of mothers and improve the outcome. It is widely reported that employers’ knowledge regarding benefits of breastfeeding is vital to understand and support breastfeeding practices at work, however, much of the previous researches have focused largely on the mothers’ perspective, hence, there is inadequate knowledge on employers’ perspective. A study by Susan Polston revealed that employer knowledge about the benefits of breastfeeding is one of the important contributing factors in deciding the employer’s role in supporting breastfeeding [29]. The knowledge of the employer about the benefits of breastfeeding usually drives the culture of the organization. Almost 1/3 of respondents in our study also perceived supporting breastfeeding is a religious obligation. Islam is the major religion of Pakistan and the Islamic guideline encourages mothers to nurse their babies for extended periods (up to 2 years) [30]. The religious affiliation could be considered while designing and promoting workplace lactation programs and policies in Pakistan. Some of the employers have a negative perception with regards to workplace support for breastfeeding i.e.; consumption of time allocated for work (26 %), financial burden on organization (22 %) and some believe it is against organizations’ decorum. Despite knowing the health benefits of breastfeeding for the baby and mothers, the employers were reluctant to invest in breastfeeding as an additional and unnecessary investment. However, many previous studies have highlighted the dual benefits of a breastfeeding friendly environment (employees and employers) [10–12]. The USA based study recommended “The business case for breastfeeding can help to create a breastfeeding-friendly jobsite and achieve desirable return on investment” [21].

In our study, breastfeeding facilities may have been overrated by employers due to the fear of losing their reputation. Additionally, breastfeeding facilities might have been confused with other staff facilities (female common room or bathroom as a lactation room and routine breaks as breastfeeding breaks). Our findings further revealed that the non-physical facilities (breastfeeding breaks, maternity leave, breastfeeding options and task adjustment) seems better off compared to physical facilities (breastfeeding corner, refrigerator, breast milk pump and nursery). The above difference could be due to the notion that the physical facilities could possibly be verified. A significant difference (*p* < 0.001) was observed in our study between national and multinational sectors with regards to breastfeeding breaks, a breastfeeding corner, task adjustment and information provided by the employer regarding breastfeeding options. We could not find any study comparing breastfeeding support in national versus multinational sites. However, the finding in our study could be due to the fact that most of the multinational companies are owned by people from developed countries, where the breastfeeding policies exist and are properly implemented by the authorities. The other possible reason could be the higher adherence to international labour laws [15, 16] by multinational companies as compared to national companies in developing countries like Pakistan. The Maternal Benefit Ordinance of Pakistan entitles working mothers to have 12 weeks paid maternity leave only, however there is no national policy on the physical breastfeeding facilities (breastfeeding corner, jobsite crèche, breast milk storage facility etc.) or non-physical facilities (breastfeeding breaks, task adjustment, breastfeeding counselling service etc.). In our study the provision of paid maternity leave was assessed according to national policy, and the result was far better than any other breastfeeding facility. This reflects the influence of a national policy on the availability of breastfeeding facilities at workplaces. Numerous studies reveal the importance of breastfeeding friendly policies at work for breastfeeding continuation. A study in Hong Kong recommended legalising the employees’ right to breastfeed their children even when the employer or supervisor is less than supportive [20]. Hence the legalised policies protect the right of a mother to breastfeed at the place of work. Adhering to the needs of lactating mothers is best achieved when organizational infrastructure is present with a clear cut policy to address these issues [31]. These policies should also incorporate the awareness and training programs of the employers on the cost-effectiveness of a worksite investment on breastfeeding facilities (investment yields a big return), additionally in the light of our study findings, the policies could also include the religious obligation for better adherence and success of the program.

We identified a few limitations in our study. The unregistered workplaces such as shops, small health clinics and home based business were not taken into account, though very small in number, they may influence the internal and external validity of our study. Additionally, our study focused only on urban areas of Pakistan, therefore, the study results cannot be generalized to rural areas, which still represent 1/3 of the female workers [10].

The study setting, Karachi, represents the largest city in Pakistan and second largest city in the world by population size, reflecting almost every ethnic group and social class living in Pakistan. We believe the results could be generalized to the other urban areas in the country, since it is likely that the working women have similar tasks, similar working conditions, similar culture, and socioeconomic status. This study could also serve as a baseline survey in similar settings; follow up studies can be done after policy revival and running workplace lactation programs to observe the effect of interventions on workplace breastfeeding facilities. Furthermore, considering our findings as evidence, these factors can be studied in large population based surveys that include urban and rural areas to get a clear picture of breastfeeding facilities at workplaces in Pakistan.

## Conclusion

We have found little support for women to maintain breastfeeding after returning to work, and probably many women have to discontinue breastfeeding earlier than planned. Breastfeeding policies for working women should be revised, to also include awareness sessions and training programs for employers about the cost-effectiveness of worksite investment on breastfeeding and educate women about their right to practice and demand breastfeeding support.

## Abbreviations

ILO: International Labour Organization
UNICEF: United Nations Children’s Emergency Fund
WABA: World Alliance for Breastfeeding Action
WHO: World Health Organization
